# An Imaging-Based Marker to Refine Risk Stratification for Transcatheter Mitral Valve Replacement

**DOI:** 10.3390/jcm14134412

**Published:** 2025-06-20

**Authors:** Liliane Zillner, Mirjam G. Wild, Michaela M. Hell, Harald Herkner, Elmar W. Kuhn, Tanja Rudolph, Thomas Walther, Lenard Conradi, Andreas Zierer, Francesco Maisano, Marco Russo, Fabrizio Rosati, Andrea Colli, Miguel Piñón, David Reineke, Gaby Aphram, Tillmann Kerbel, Christophe Dubois, Jörg Hausleiter, Ralph Stephan von Bardeleben, Markus Mach, Christian Loewe, Martin Andreas

**Affiliations:** 1 Department of Cardiac and Thoracic Aortic Surgery, Medical University of Vienna, Währinger Gürtel 18-20, 1090 Vienna, Austria; liliane.zillner@meduniwien.ac.at (L.Z.);; 2 Medizinische Klinik I, LMU University Hospital, 80336 Munich, Germany; 3 Department of Cardiology, University Medical Center Mainz, 55131 Mainz, Germany; 4 Department of Emergency Medicine, Medical University of Vienna, 1090 Vienna, Austria; 5 Department of Cardiothoracic Surgery, Heart Center, University Hospital Cologne, 50937 Cologne, Germany; 6 Department of Cardiology, Heart and Diabetes Center Northrhine-Westfalia, Bad Oeynhausen, Ruhr University Bochum, 32545 Bochum, Germany; 7 Department of Cardiac Surgery, University Hospital Frankfurt, 60598 Frankfurt, Germany; 8 Department of Cardiac Surgery, Heart Center, University Hospital Cologne, 50937 Cologne, Germany; 9 Department for Cardiac, Vascular and Thoracic Surgery, Johannes Kepler University Linz, Kepler University Hospital, 4020 Linz, Austria; 10 Valve Center, IRCCS Ospedale San Raaffaele and University Vita Salute, 20132 Milano, Italy; 11 Department of Cardiac Surgery and Heart Transplantation, Azienda Ospedaliera San Camillo Forlanini, 00152 Rome, Italy; 12 Division of Cardiac Surgery, Spedali Civili di Brescia, University of Brescia, 25123 Brescia, Italy; 13 Department of Surgical, Medical and Molecular Pathology and Critical Care Medicine, University of Pisa, 56124 Pisa, Italy; 14 Servicio Cirugía Cardíaca, Hospital Álvaro Cunqueiro, 36312 Vigo, Spain; 15 Department of Cardiac Surgery, Inselspital University Hospital Bern, 3010 Bern, Switzerland; 16 Department of Cardiovascular and Thoracic Surgery, Cliniques Universitaires Saint Luc, 1200 Brussels, Belgium; 17 Department of Cardiovascular Medicine, University Hospital Leuven and Department of Cardiovascular Sciences, 3000 Leuven, Belgium; 18 DZHK (German Center for Cardiovascular Research), Partner Site Munich Heart Alliance, 80636 Munich, Germany; 19 Division of Cardiovascular and Interventional Radiology, Department of Biomedical Imaging and Image-Guided Therapy, Medical University of Vienna, 1090 Vienna, Austria

**Keywords:** transcatheter mitral valve replacement (TMVR), left ventricular end-diastolic diameter index (LVEDDi), Tendyne™ valve system

## Abstract

**Background**: The Tendyne™ transcatheter heart valve (THV) system is a promising option for high-risk patients with severe mitral regurgitation (MR) who are ineligible for surgery or transcatheter edge-to-edge repair (TEER). As most fatal complications occur within the first 90 days, this study aimed to identify anatomical predictors of in-hospital mortality after transcatheter mitral valve replacement (TMVR). **Methods:** In this subanalysis of the TENDER registry, data from 110 patients who underwent TMVR across 26 centers between January 2020 and June 2022 were evaluated. Preprocedural imaging parameters were analyzed, including transthoracic echocardiography (TTE), transesophageal echocardiography (TEE), and cardiac 4D computed tomography (CT). **Results:** We identified LVEDDi as a significant predictor of in-hospital mortality (*p* = 0.022), with lower values in non-survivors (26.42 ± 3.76 mm/m^2^) than in survivors (30.37 ± 5.58 mm/m^2^). Both indexed and absolute LVEDDi predicted in-hospital complications (*p* < 0.001 and *p* = 0.008). In multivariate analysis, LVEDDi (*p* = 0.048; OR = 0.856) and STS score (*p* = 0.038; OR = 1.114) remained independent predictors of in-hospital mortality. In an extended model, only LVEDDi persisted as a significant predictor (*p* = 0.007), highlighting its robustness. **Conclusions**: This analysis identified a small LVEDDi as a novel, clinically relevant risk factor in TMVR and showed its added value alongside conventional markers. Its easy calculation supports incorporating LVEDDi thresholds into screening to improve patient selection and outcomes.

## 1. Introduction

**Figure 1:**
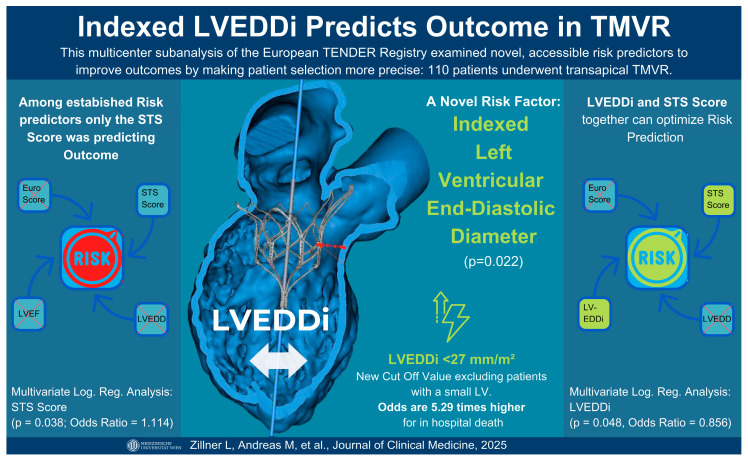
Visual Abstract: Indexed LVEDDi predicts Outcome in TMVR. LVEDDi, Left Ventricular End-Diastolic Diameter Index; STS Score: Society of Thoracic Surgeons Score; LV, Left Ventricle; LVEF, Left Ventricular Ejection Fraction; EuroSCORE, European System for Cardiac Operative Risk Evaluation; TMVR; Transcatheter Mitral Valve Replacement. Adapted with permission from Abbott. Created using Canva.

The Tendyne™ transcatheter heart valve (THV; Abbott Cardiovascular, Plymouth, MN, USA) is currently the only Conformité Européenne (CE)-marked TMVR device commercially available in Europe. It is designed for patients with severe symptomatic mitral regurgitation (MR) who are at high or prohibitive surgical risk and are not suitable candidates for transcatheter edge-to-edge repair (TEER). TEER ineligibility often results from anatomical challenges—such as large coaptation gaps, severe leaflet tethering, mitral annular calcification, or complex valve anatomy—or from advanced left ventricular (LV) dysfunction, where durable repair is unlikely [1,2,3,4] (Figure 1).

The THV is delivered via a transapical approach and anchored with a tether to an epicardial pad, providing stable fixation within the native mitral annulus. Its design enables controlled deployment and complete valve replacement, making it particularly suitable for patients with functional MR (FMR) or anatomies where leaflet grasping is not feasible or would result in residual regurgitation [1,2,3,4].

First, real-world data on the THV valve system from a European multicenter experience (TENDyne European expeRience registry study) revealed promising technical and procedural success rates of 96% and 80%, respectively, leading to the elimination of MR in most patients [5,6]. Nevertheless, TMVR faces unique challenges, including left ventricular outflow tract obstruction (LVOTO) and complications related to apical access. With most of these fatal complications occurring within the first 90 days after intervention, recent studies have reported a higher 30-day mortality rate with TMVR compared to TEER, likely reflecting the more complex risk profile of this patient population [1,7].

A small LV, whether due to an anatomically small heart or low preload, may predispose to complications commonly associated with TMVR. First, geometric or dynamic LVOTO may be exacerbated by the close proximity of the MV to the septum in small hearts or transiently in low-volume states, as seen in patients with hypertrophic obstructive cardiomyopathy (HOCM) or hypotensive critical illness [8]. Second, reduced preload may decrease MV tethering, further facilitating LVOTO [9]. Similarly, in the setting of volume depletion due to major bleeding, a common cause of periprocedural death in TMVR, a small LV may promote rapid circulatory collapse due to its limited volume reserve and rapid emptying, followed by terminal cardiac decompensation [10]. This study aimed to investigate whether preprocedural imaging parameters, with a focus on the echocardiographic assessment of a small LV, can predict in-hospital mortality among patients undergoing TMVR.

## 2. Materials and Methods

### 2.1. Study Population

**Figure 2:**
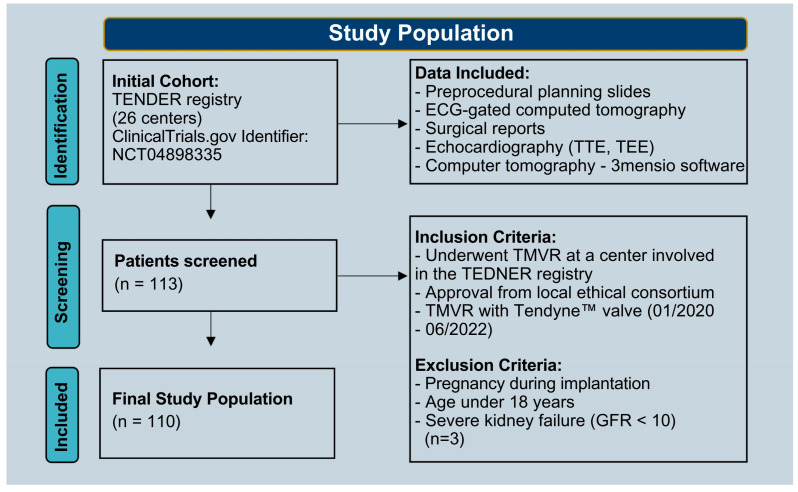
Flow chart indicating the identification of the study population, including the inclusion and exclusion criteria.

This subanalysis of the TENDER register included data from 26 centers and aimed to identify significant predictors of in-hospital mortality and complications among patients undergoing TMVR (ClinicalTrials.gov identifier: NCT04898335). Our dataset consisted of preprocedural planning slides, electrocardiogram (ECG)-gated computed tomography, and surgical reports of patients treated with TMVR between January 2020 and June 2022. Patients were excluded if they were pregnant, younger than 18 years old, or had severe preoperative kidney failure (GFR < 10). The latter criterion was applied because dialysis could confound volume management and imaging parameters. The bleeding grades were determined based on the Mitral Valve Academic Research Consortium (MVARC) [11]. Data from 110 patients were analyzed (Figure 2).

### 2.2. Preintervention Imaging

In accordance with the imaging requirements for preprocedural anatomical assessment, transthoracic echocardiography (TTE), transesophageal echocardiography (TEE), low-dose chest CT, and retrospectively ECG-synchronized, contrast-enhanced cardiac CT spanning the entire cardiac cycle and heart were performed. In addition to these mandatory imaging modalities, further parameters were measured using 3mensio software (V10.3, Pie Medical Imaging BV, Maastricht, The Netherlands), including the diameter of the apex and the length of the anterior mitral valve leaflet (AMVL).

**Figure 3:**
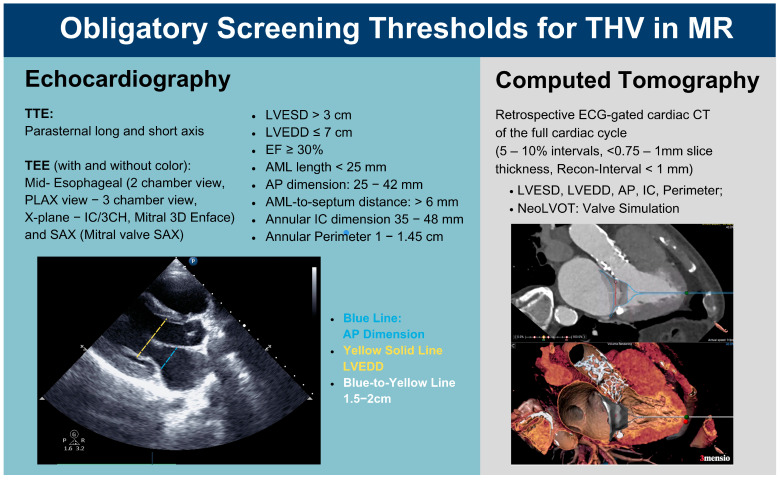
Obligatory screening thresholds for THV on MR. AML, Anterior Mitral Leaflet; AP, Anterior–Posterior; CT, Computed Tomography; EF, Ejection Fraction; IC, Intercommissural; IC/3CH, Intercommissural 3 Chamber View; LVEDD, Left Ventricular End-Diastolic Diameter; LVESD, Left Ventricular End-Systolic Dimension; MR, Mitral Regurgitation; NeoLVOT, Neo Left Ventricular Outflow Tract; PLAX, Parasternal Long-Axis; SAX, Short-Axis; TTE, Transthoracic Echocardiography; TEE, Transesophageal Echocardiography; THV, Transcatheter Heart Valve (Abbott Prescreening Guides based on [12,13,14], created unsing Canva).

To optimize patient selection, we focused on refining and complementing anatomical thresholds within the preintervention screening recommendations. For TMVR with an apical tethered valve, patients were required to meet specific anatomical criteria for prosthesis anchoring and risk mitigation. These thresholds (Figure 3) address factors such as systolic anterior motion (SAM), LVOTO, and LV collapse. Notably, the LVEDDi was evaluated during preintervention screening via TTE; however, a recommended minimum indexed diameter has not yet been established.

### 2.3. Statistical Analysis

Baseline characteristics were compared between survivors and non-survivors in a case-control design. Continuous variables are reported as mean ± standard deviation or median with interquartile range. Group comparisons and correlations were performed using standard parametric or non-parametric tests. Univariate and multivariate logistic regression identified predictors of in-hospital mortality. A spline curve illustrated the non-linear association between LVEDDi and mortality.

## 3. Results

Of 110 TMVR patients (mean age 75), 12 died in hospital. LVEDDi was lower in non-survivors (26.42 ± 3.76 mm/m^2^) vs. survivors (30.37 ± 5.58 mm/m^2^; *p* = 0.019). Absolute LVEDD was also lower but not statistically significant. Univariate analysis showed the STS score predicted outcomes (*p* = 0.02), while EuroSCORE II did not. Most patients were NYHA class III (*n* = 83), followed by class II (*n* = 18) and IV (*n* = 9); none of the class II patients died. Heart failure hospitalization occurred in 91% of non-survivors vs. 63% of survivors. Baseline imaging showed trends toward smaller diastolic neoLVOT, lower LA height, reduced A2 clearance, and slightly shorter AMVL (Table 1).

### 3.1. Predictors of In-Hospital Mortality

Univariate logistic regression revealed that LVEDDi (*p* = 0.022) was the only significant imaging-based predictor of mortality, while the STS score (*p* = 0.04) was also a significant clinical predictor (Table 2).

**Figure 4:**
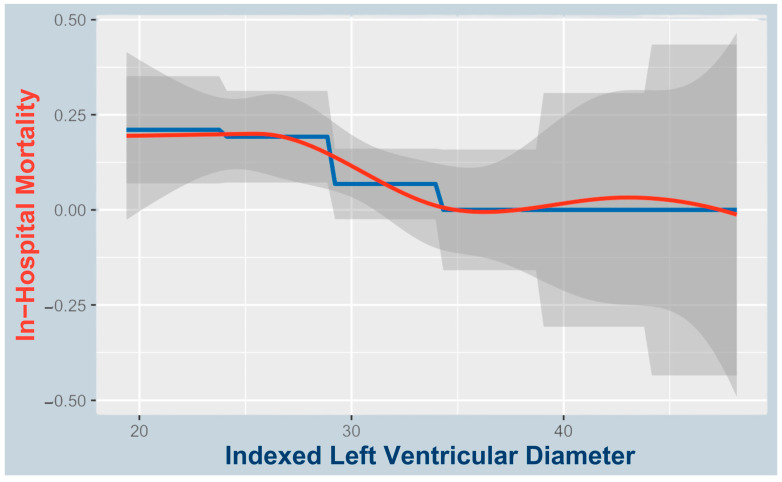
Natural spline curve: LVEDDi and in-hospital mortality. A natural spline model analyzing the relationship between the LVEDDi and in-hospital mortality, including three knots (26, 34, and 42), with boundary knots at 25 and 75 (adjusted R^2^ = 0.0257). Blue line: actual observed data representing the probability of in-hospital death; red line: smoothed spline values showing the modeled trend.

A proposed cutoff of 27 mm/m^2^ identified higher in-hospital mortality below this threshold (8/35; 22.9%) vs. ≥27 mm/m^2^ (4/75; 5.3%; see Figure 4). The odds ratio for in-hospital mortality below the proposed LVEDDi cutoff was 5.29 (95% CI: 1.53–18.23). All patients with fatal access complications (*n* = 3; 2 procedural, 1 at 30 days) had LVEDDi < 27 mm/m^2^.

### 3.2. Causes of Death

Of the 12 deaths, 4 were procedural: 3 from ventricular rupture with fatal bleeding and 1 from LVOTO. Among nine patients who died within 30 days, causes included major access complications with fatal bleeding (*n* = 1), bleeding with sepsis (*n* = 1), sepsis with acute kidney failure (*n* = 1), bleeding with kidney failure (*n* = 1), and kidney failure alone (*n* = 1). Three in-hospital deaths beyond 30 days were due to sepsis (*n* = 2) and kidney failure (*n* = 1).

### 3.3. LVEDDi’s Correlation to the STS Score

Multivariate regression showed LVEDDi (*p* = 0.048; OR = 0.856, CI: 0.734–0.999) and STS score (*p* = 0.038; OR = 1.114, CI: 1.006–1.235) as significant predictors. Pearson correlation between them was weak (r = −0.026; *p* = 0.794). In an extended model, only LVEDDi remained significant (*p* = 0.007), highlighting its independent prognostic value.

### 3.4. Predictors of In-Hospital Complications

LVEDDi was a significant predictor of in-hospital complications (*p* < 0.001; Table 3). Patients with complications (*n* = 66) had lower LVEDDi (26.42 ± 3.76) vs. those without (*n* = 43; 32.10 ± 6.14 mm^2^/m^2^). LVEDD was also lower (53.16 ± 7.87 vs. 57.34 ± 8.06 mm; *p* = 0.008). Logistic regression confirmed inverse associations for LVEDDi (OR = 0.867; *p* < 0.001) and LVEDD (OR = 0.929; *p* = 0.006).

Key complications associated with in-hospital mortality were major access issues (*n* = 8), bleeding grades 2–5 (*n* = 23), sepsis (*n* = 11), ECMO (*n* = 4), and LVOTO (*n* = 9) (Table 4).

Smaller LVEDDi correlated with smaller neoLVOT and A2 clearance, potentially contributing to LVOTO. Significant Spearman correlations were found for systolic neoLVOT (r = 0.242; *p* = 0.012), systolic A2 clearance (r = 0.202; *p* = 0.043), diastolic neoLVOT (r = 0.265; *p* = 0.008), and diastolic A2 clearance (r = 0.307; *p* = 0.002).

### 3.5. Associations with AF and LVEDDi at Baseline

In the baseline cohort, LVEDDi was a significant predictor of atrial fibrillation (AF). Logistic regression showed that lower LVEDDi values were associated with a higher incidence of AF (B = −0.087, *p* = 0.023; OR = 0.916), indicating that smaller ventricular chamber size contributes to AF development.

In contrast, the non-indexed LVEDD also showed a trend toward significance (B = −0.047, *p* = 0.062; OR = 0.954), suggesting a similar inverse relationship with AF risk. However, this result did not reach conventional statistical significance, reinforcing the added value of body surface area indexing.

Furthermore, both left ventricular ejection fraction (LVEF) (*p* = 0.003; OR = 1.063) and NYHA functional class (*p* = 0.027; OR = 2.628) were independently associated with AF. There was a near-significant association for left atrial height measured on CT planning slides (*p* = 0.082; OR = 1.040), suggesting a potential relationship between atrial remodeling and AF. In contrast, other variables such as NT-proBNP (*p* = 0.729), mitral regurgitation grade (*p* = 0.411), and the STS score (*p* = 0.085) showed no statistically significant association with AF.

## 4. Discussion

Recent studies have demonstrated improvements in TMVR outcomes with increasing institutional experience. Two earlier analyses reported 30-day mortality rates of 23.5% and 17%, respectively. In the first study by Wilde et al., which included 63 patients eligible for Tendyne, TEER was performed when feasible. The 30-day mortality rate was higher in the Tendyne group (*n* = 17) compared to the TEER group (*n* = 46). The authors also evaluated reverse remodeling and mitral regurgitation reduction. In the second study, analyzing the TENDER registry, Hell et al. reported a 1-year cardiovascular mortality rate of 17% and successful MR reduction to ≤1+ in 87% of 195 enrolled patients. Our subanalysis reflects this trend, with a 30-day mortality rate of 8.2% [7,15,16]. Despite these advancements, TMVR mortality remains high, with most fatal complications, such as LVOTO and bleeding, occurring within 90 days [1]. Recent analyses have investigated various predictors of outcomes and aspects of LV remodeling. One study (*n* = 191) identified age, pulmonary hypertension, and institutional experience as independent predictors of short-term mortality. Another analysis (*n* = 127) found that patients with fatal access complications tended to have thinner apical myocardium, suggesting that such patients may be suboptimal candidates for apical TMVR. A third study (*n* = 36) linked favorable LV remodeling at one month post-TMVR, assessed by CT angiography, to apical pad positioning [10,16,17]. Although our study included a modest cohort size (*n* = 110), it is among the largest to propose an approach incorporating a novel selection criterion—namely, a small LVEDDi—for risk prediction and potential mortality reduction.

### 4.1. Small LVEDDi as a Predictor of In-Hospital Mortality and Complications

This study outlines the predictive value of the LVEDDi—indicative of a small ventricle—for in-hospital mortality and complications among patients undergoing TMVR (Figure 1). According to the best practice recommendations for Tendyne by Duncan et al., on the basis of a multicenter analysis and prescreening guides (Figure 2), a threshold for a low LVEDDi is not currently listed but should probably be considered a novel risk and selection criterion for major complications, especially since it can be easily integrated into routine preprocedural screening [1,7,18,19,20].

Patients with a smaller LVEDDi presented a greater risk of in-hospital death. While the mean LVEDDi in survivors was 30.37 ± 5.58 mL/m^2^, those who died had a lower mean of 26.42 ± 3.76 mL/m^2^ (*p* = 0.022). Thus, a reduced LVEDDi may reflect pathological ventricular remodeling and hypertrophy, impaired cardiac function, or a high risk for LVOTO due to dynamic systolic anterior motion (SAM) of the AMVL.

Nonetheless, our proposed LVEDDi cutoff of <27 mm/m^2^ is data-driven; however, further studies are needed to validate this threshold, given the limited sample size. Additionally, related markers such as left ventricular end-diastolic volume indexed to body surface area (LVEDVi) or the left atrioventricular coupling index may warrant further investigation [21].

### 4.2. LVEDDi as Indicator of AF at Baseline

Our findings, identifying a lower indexed LVEDD as a significant predictor of AF, align closely with the review by Mauriello et al., which analyzed approximately 4000 patients. This review emphasized the role of reduced left atrial strain (LAS), a marker of indexed LA tissue deformation, in early atrial dysfunction and AF development [22]. Our data suggest that a relatively small, indexed left ventricle may limit preload reserve, thereby increasing left atrial pressure and stretch. Notably, non-indexed LVEDD showed only a borderline association with AF, reinforcing the importance of indexing to body surface area.

Importantly, MR severity was not significantly associated with AF in our cohort, underscoring the multifactorial nature of MR. Beyond regurgitation severity, atrial mechanical load and its interaction with diastolic dysfunction appear more relevant. In patients with small, stiff ventricles and impaired compliance, the loss of atrial contraction due to AF may significantly impair ventricular filling. AF may, therefore, arise as a consequence of increased atrial strain from a small ventricle or contribute to impaired preload and progressive remodeling itself.

The findings of Mauriello et al. reinforce this interconnectedness of LA and LV function by demonstrating that LAS, similar to LVEDDi, serves as an imaging marker of both diastolic and atrial function [22]. Together, these insights underscore the tight coupling between atrial and ventricular structure and function, which is critical for individualized risk stratification in candidates for TMVR.

### 4.3. Pathophysiologic Considerations Regarding a Smaller LVEDDi and Increased Mortality

#### 4.3.1. Rapid Hemodynamic Compromise in Access Complication or Bleeding

Acute hemodynamic compromise due to bleeding or access complications occurred primarily intraoperatively or in the immediate periprocedural period. Among the eight patients with access complications, four died—three during the procedure and one within 30 days. In three of these four cases, procedural timelines and clinical context strongly suggest that rapid volume loss led to circulatory collapse, particularly in the setting of a small LV. Notably, all three had an LVEDDi below the proposed cutoff of 27 mm/m^2^ and had undergone successful valve implantation, highlighting the potential vulnerability of patients with smaller ventricles. The fourth patient, whose LVEDDi was above the cutoff, did not reach technical success (valve implantation) and represents a rare and particularly complex case within the context of a 96% overall technical success rate reported in TMVR registries [5,6]. Furthermore, access site complications (*p* < 0.001) and bleeding grades 2, 3, or 5 (*p* = 0.004) significantly contributed to in-hospital mortality, underscoring the clinical impact of intraprocedural bleeding. These findings support the potential role of LVEDDi as a risk stratification marker and suggest that bleeding may be particularly detrimental in patients with small ventricles, which may empty more rapidly and decompensate earlier in the setting of acute volume loss.

#### 4.3.2. Diastolic Dysfunction and Hypertrophy

A small and hypertrophied ventricle is less compliant and difficult to fill adequately. Diastolic dysfunction, which leads to lower stroke volumes and cardiac output, impacts the ability to maintain adequate coronary or organ perfusion pressures [23]. Moreover, the hypertrophied myocardium may also suffer from compromised microcirculation [24,25].

Furthermore, a small LVEDDi may reflect a maladaptive, possibly concentric remodeling phenotype in selected patients, which may represent an atypical and potentially deleterious response in the setting of chronic MR [26].

### 4.4. LVEDDi and Its Correlation with the STS Score

The STS score is a widely used risk assessment tool that estimates the probability of mortality and major complications for patients undergoing conventional open chest cardio-thoracic surgeries and could be verified for minimally cardiac procedures according to our data [27]. We propose considering the LVEDDi specifically for TMVR interventions, as it provides valuable information in addition to the STS score. While the neglectable negative correlation (r = −0.026, *p* = 0.026) demonstrated that the STS score and LVEDDi are independent predictors and do not correlate with each other, adding the LVEDDi to the risk assessment for TMVR procedures would increase the accuracy of mortality prediction. Although an absolute LVEDD cutoff is already integrated into current preprocedural screening protocols, the indexed LVEDDi has not yet been considered—potentially underestimating its superior accuracy, especially in patients with non-standard anatomy. The LVEDDi provides a more accurate, patient-specific assessment.

### 4.5. What the Indexed LVEDDi Captures That the Absolute LVEDD Misses

By nature, the LVEDDi accounts for anatomical variation by adjusting for BSA (females: 23–31 mm/m^2^, males: 22–30 mm/m^2^) [28]. Therefore, relatively smaller ventricular sizes—particularly in larger individuals, often men—can be more accurately identified. In such patients, the absolute LVEDD may still fall within sex-specific normal ranges yet be disproportionately small when indexed to BSA, potentially indicating impaired preload reserve relative to circulating blood volume and metabolic demand. This is supported by a cross-sectional study reporting lower LVEDDi values in men despite women exhibiting smaller absolute LVEDD measurements [29].

By contrast, the LVEDDi may provide a more accurate, patient-specific assessment in smaller individuals—often female—too, where a small absolute LVEDD might be normal rather than indicative of hypertrophy or stiffening.

Overall, the LVEDDi may more effectively identify high-risk patients with small ventricular sizes relative to their BSA and, consequently, blood volume, thereby better capturing those susceptible to a mismatch between cardiac output and metabolic demand. Their limited hemodynamic reserve may promote complications such as bleeding or LVOT obstruction. Thus, the application of LVEDDi in preprocedural planning might be an easily implementable adjustment, not only improving patient selection but also enhancing inclusiveness within the spectrum of high-risk MR patients.

This could be facilitated during obligatory planning slide assessment, as LVEDD is part of the current screening scheme. Adjustment for BSA to calculate LVEDDi should be undertaken and incorporated into the risk assessment on planning slides provided to the heart team and surgeon, preferably with highlighting of whether the LVEDDi falls below or above the proposed cutoff.

### 4.6. A Small Ventricle Might Contribute to Late-Onset SAM

LVOTO is one of the most common complications in TMVR, with in-hospital mortality rates of up to 62% [2]. Despite stringent criteria in patient selection and specific requirements for ventricular size, this analysis confirmed that peri-interventional LVOTO remains a significant contributor to in-hospital death (*n* = 3/12, *p* = 0.025). On the basis of our data, there is reason to believe that a reduced LVEDDi in an already preselected patient cohort may correlate with a greater risk for LVOTO in the context of a delayed appearance [8]. Postoperatively, remodeling induced by the competent novel valve may further narrow the LVOT, potentially triggering late-onset SAM and subsequent LVOTO in patients with small ventricles, as a low LVEDDi correlates with a smaller neo-LVOT in our cohort [17]. Furthermore, LVEDDi is measured in the parasternal long-axis view, where the anterior–posterior mitral leaflet distance is visualized. A small LVEDDi may reflect a similarly short leaflet distance, potentially favoring late-onset SAM. While geometric SAM results from valve implantation-induced push of the AMVL, which further narrows the LVOT, dynamic SAM may be triggered by increased blood flow through the narrowed LVOT (due to Bernoulli forces). Additionally, volume depletion, which occurs more rapidly in a small LV, leads to a decrease in MV tethering and the EDV, further narrowing the LVOT and subsequently increasing the flow velocity, potentially triggering a dynamic SAM [9]. This is typical for sepsis, with a significant contribution to in-hospital death (*n* = 6, *p* < 0.001) [30]. It not only leads to tachycardia but also leads to decreased effective circulating blood volume when it tends toward distributive shock. We hypothesize that both increased heart rate and volume depletion increase the flow speed through the LVOT, further contributing to a pressure differential that pulls the AMVL toward the LVOT, causing SAM and, ultimately, LVOTO. With potential late-onset SAM in focus, the technique of AMVL splitting should be further explored.

### 4.7. Future Outlook

As transseptal TMVR technologies continue to evolve, the risk of access-related complications, particularly apical bleeding exacerbated by a small LV, may become a concern of the past [31]. However, other procedure-specific risks, such as LVOTO, remain relevant, as the AML is retained even when the valve is delivered via a transseptal approach. Therefore, the insights gained from this study on the association between LV size and in-hospital mortality in TMVR may remain relevant for future device iterations, including those employing alternative access routes, as a small LV may continue to influence procedural safety, hemodynamic tolerance, and anatomical feasibility regardless of delivery approach.

The incorporation of a low LVEDDi as a novel risk marker for high-risk MR patients may represent significant progress in reducing the mortality rate of TMVR patients, particularly as these patients currently have no alternative treatment options. Future studies, including follow-ups of the TENDER and similar registries with larger cohorts, are warranted to further explore and confirm the role of LVEDDi in short- and long-term outcomes and to specify subgroup effects related to MR etiology or cause of death—particularly in the context of this highly selected and morbid patient cohort. The role of LVEDDi in TEER and transseptal-access TMVR may also represent a relevant focus for future scientific investigation.

Limitations of this study include its retrospective design, which precludes causal inference and introduces the potential for selection and information bias—particularly across multiple centers with variable imaging protocols and clinical practices. Although standardized data collection was pursued, inconsistencies in imaging acquisition, documentation, and missing data may have affected measurement reliability and internal validity. The sample size (*n* = 110), though one of the larger TMVR cohorts, limits statistical power for mortality and subgroup analyses, increasing the risk of Type II error and limiting multivariable adjustments. Finally, generalizability is limited by the inclusion of a high-risk, highly selected population treated with the Tendyne™ system. Findings may not extend to other TMVR devices or lower-risk patients. Prospective multicenter studies are needed to validate LVEDDi as a screening tool across broader settings.

Given the extreme multimorbidity and frailty of our study cohort, which reflects considerable heterogeneity, future studies with larger sample sizes are warranted to enable subgroup analyses (e.g., by MR etiology or gender). Such investigations may further refine risk stratification and help validate our findings in more defined patient populations.

## 5. Conclusions

This study demonstrated that a small LVEDDi is a significant and novel predictor of in-hospital mortality in TMVR patients. Given its superiority over conventional risk markers, especially when used in combination with the STS score, the LVEDDi might help clinicians better identify high-risk patients, enhance procedural planning, and ultimately improve patient outcomes.

## Figures and Tables

**Table 1:** Baseline Characteristics.

Baseline Characteristics	In-Hospital Survival (*n* = 98)	In-Hospital Death (*n* = 12)	*p*-Value
BMI (kg/m^2^)	26.15 ± 5.07	27.09 ± 3.28	0.53
Age (years)	77 (12)	79.5 (12)	0.23
Male	54 (55.1)	8 (66.67)	0.45
Female	44 (44.9)	4 (33.33)	0.45
EuroScore II (%)	6.3 (6.8)	8.45 (7.08)	0.16
STS	5.8 (6.4)	7.65 (10.03)	0.02
NT-pro-BNP (pg/mL)	3578 (5374)	3843 (11392)	0.94
GFR (mL/min)	42 (31)	37.5 (19.8)	0.14
sPAP (mmHg)	51.68 ± 14.76	58.27 ± 18.51	0.18
NYHA Class II	18 (18.37)	0	0.26
NYHA Class III	72 (73.47)	11 (91.67)	0.26
NYHA Class IV	8 (8.16)	1 (8.33)	0.26
Heart Failure Hospitalization	60 (62.5)	10 (90.9)	0.06
Atrial Fibrillation	62 (63.27)	7 (58.33)	0.74
COPD	16 (16.33)	4 (33.33)	0.15
Prior Stroke	14 (14.29)	1 (8.33)	0.57
Coronary Artery Disease	59 (60.2)	10 (83.33)	0.12
Prior Myocardial Infarction	17 (17.53)	4 (33.33)	0.19
Prior Mitral Valve Intervention	9 (9.18)	0	0.27
Prior Mitral Valve Surgery	3 (3.06)	0	0.54
Primary MI	41 (41.84)	5 (41.67)	0.97
Secondary MI	35 (35.71)	4 (33.33)	0.97
Mixed MI	22 (22.45)	3 (25.00)	0.97
Echo + CT Baseline			
2D Echocardiographic Parameters:			
LVEF (%)	50 (17)	50 (19)	0.17
LVEDD (mm)	55.28 ± 8.06	50.83 ± 8.04	0.07
LVEDDi (mm/m^2^)	30.37 ± 5.58	26.42 ± 3.76	0.02
TAPSE < 1 (cm)	33 (35.87)	4 (36.36)	0.97
Tricuspid Regurgitation III–IV	23 (23.71)	5 (41.67)	0.18
(ECG Gated) 2D-CT Parameters:			
Diameter of the Apex (mm)	5.89 ± 2.09	5.33 ± 2.18	0.38
Apex to Surgical Apex (mm)	2.5 (2.2)	2.5 (1.6)	0.71
LAD Distance (mm)	17.24 ± 8.45	17.18 ± 9.62	0.98
Ant.-Post. Oversize (%)	6 (7.7)	7.8 (19.5)	0.26
Intercommissural Oversize (%)	18.2 (8.5)	13 (8.3)	0.41
A2 Clearing Systole (mm)	10.6 (5.35)	11.8 (1.7)	0.63
End systolic Neo-LVOT (mm^2^)	380 (169.65)	364.2 (30.85)	0.88
A2 Clearing Diastole (mm)	11.7 (6.4)	9.6 (6.95)	0.35
End diastolic Neo-LVOT (mm^2^)	431.5 (228)	310 (215.4)	0.26
AMVL length (mm)	21.7 (4.3)	20.6 (9.9.)	0.42
Left Atrium height (mm)	67 ± 9.78	64.68 ± 9.6	0.44

STS score = Society of Thoracic Surgeons score; GFR = glomerular filtration rate; NT-proBNP = B-type natriuretic peptide; sPAP = systolic pulmonary pressure; COPD = chronic obstructive pulmonary disease); LVEDD = left ventricular end-diastolic diameter; LVEDDi = indexed left ventricular end-diastolic diameter; LEF = left ejection fraction; LAD distance = distance from the apical access point to the left descending artery; AMVL length = Anterior Mitral Leaflet Length.

**Table 2:** The Parameter Defining a Small Ventricle as a Predictor of In-Hospital Death.

	B	S.E.	*p*-Value	Exp(B) (Odds Ratio)	95% CI for Exp(B)
Clinical Characteristics					
EuroSCORE II (%)	0.029	0.045	0.515	1.030	0.943–1.124
STS Score (%)	0.110	0.052	0.036	1.116	1.007–1.236
NT-pro-BNP (pg/mL)	0.000	0.000	0.907	1.000	1.000–1.000
GFR (mL/min)	−0.024	0.017	0.159	0.976	0.943–1.010
sPAP (mmHg)	0.026	0.020	0.181	1.027	0.988–1.067
2D Echocardiographic Parameters:					
LVEF (%)	−0.037	0.029	0.209	0.964	0.91–1.021
LVEDD (mm)	−0.073	0.042	0.080	0.930	0.857–1.009
LVEDDI (mm/m^2^)	−0.159	0.069	0.022	0.853	0.745–0.977

Logistic regression was applied to identify parameters that predict in-hospital death. CI = confidence interval; S.E. = standard error.

**Table 3:** Parameters Constituting a Small Ventricle as Independent Predictors for In-Hospital Complications (*t*-test, log regression).

*t*-Test:	No In-Hospital Complications (*n* = 43)	In-Hospital Complications (*n* = 66)	*p*-Value
LVEDD	57.34 ± 8.06	53.16 ± 7.87	0.008
LVEDDi (mm/m^2^)	32.10 ± 6.14	26.42 ± 3.76	<0.001
**Logistic Regression:**		OR (95%-CI)	*p*-value
LVEDD		0.929 (0.881–0.975)	0.006
LVEDDi (mm/m^2^)		0.867 (0.796–0.946)	<0.001

LVEDD = left ventricular end-diastolic diameter; LVEDI = indexed left ventricular end-diastolic diameter.

**Table 4:** Major In-Hospital Complications and their impact on In-Hospital Mortality.

Major Complications	Total (*n*)	In-Hospital Mortality (*n*)	*p*-Value
Patients	110	10	
Major Access Complication (= Life-Threatening Bleeding)	8	4	<0.001
Bleeding grade 2, 3, or 5	23	6	0.004
Sepsis	11	6	<0.001
ECMO	4	3	<0.001
Stroke			
-nondisabling	2	1	0.211
-disabling	1	0	
LVOTO	9	3	0.025
Acute Kidney Injury	23	5	0.011
Acute Dialysis	2	1	0.035
Reintervention (any cause)	4	0	0.46
Full Sternotomy	1	1	0.004
Myocardial Infarction	1	0	0.76

Major complications in TMVR patients and their impact on in-hospital death. Bleeding grades were determined based on the Mitral Valve Academic Research Consortium (MVARC) Primary Bleeding Scale. LVOTO = left ventricular outflow tract obstruction; ECMO = extracorporeal membrane oxygenation.

## Data Availability

No new data were created or analyzed in this study. Data sharing is not applicable to this article.

## Abbreviations

The following abbreviations are used in this manuscript:

AML	Anterior Mitral Leaflet
AP	Anterior–Posterior
BSA	Body Surface Area
CI	Confidence Interval
COPD	Chronic Obstructive Pulmonary Disease
CT	Computed Tomography
ECG	Electrocardiogram
ECMO	Extracorporeal Membrane Oxygenation
EF	Ejection Fraction
GFR	Glomerular Filtration Rate
IC	Intercommissural
LA	Left Atrium
LV	Left Ventricle
LVEDD	Left Ventricular End-Diastolic Diameter
LVEDDi	Indexed Left Ventricular End-Diastolic Diameter
LVESD	Left Ventricular End-Systolic Diameter
LVOT	Left Ventricular Outflow Tract
LVOTO	Left Ventricular Outflow Tract Obstruction
MVARC	Mitral Valve Academic Research Consortium
MR	Mitral Regurgitation
MV	Mitral Valve
NeoLVOT	Neo Left Ventricular Outflow Tract
NYHA	New York Heart Association
OR	Odds Ratio
SAM	Systolic Anterior Motion
STS	Society of Thoracic Surgeons
TEE	Transesophageal Echocardiography
TEER	Transcatheter Edge-to-Edge Repair
THV	Transcatheter Heart Valve
TMVR	Transcatheter Mitral Valve Replacement
TTE	Transthoracic Echocardiography
